# E-cadherin Polarity Is Determined by a Multifunction Motif Mediating Lateral Membrane Retention through Ankyrin-G and Apical-lateral Transcytosis through Clathrin[Fn FN1]

**DOI:** 10.1074/jbc.M113.454439

**Published:** 2013-03-25

**Authors:** Paul M Jenkins, Chirag Vasavda, Janell Hostettler, Jonathan Q. Davis, Khadar Abdi, Vann Bennett

**Affiliations:** From the ‡ Howard Hughes Medical Institute and; § Departments of Biochemistry,; ¶ Cell Biology, and; ‖ Neurobiology, Duke University Medical Center, Durham, North Carolina 27710

**Keywords:** Cell Adhesion, Cell Biology, Cell Junctions, Cell Polarity, Molecular Cell Biology

## Abstract

We report a highly conserved motif in the E-cadherin juxtamembrane domain that determines apical-lateral polarity by conferring both restricted mobility at the lateral membrane and transcytosis of apically mis-sorted protein to the lateral membrane. Mutations causing either increased lateral membrane mobility or loss of apical-lateral transcytosis result in partial mis-sorting of E-cadherin in Madin-Darby canine kidney cells. However, loss of both activities results in complete loss of polarity. We present evidence that residues required for restricted mobility mediate retention at the lateral membrane through interaction with ankyrin-G, whereas dileucine residues conferring apical-lateral transcytosis act through a clathrin-dependent process and function in an editing pathway. Ankyrin-G interaction with E-cadherin is abolished by the same mutations resulting in increased E-cadherin mobility. Clathrin heavy chain knockdown and dileucine mutation of E-cadherin both cause the same partial loss of polarity of E-cadherin. Moreover, clathrin knockdown causes no further change in polarity of E-cadherin with dileucine mutation but does completely randomize E-cadherin mutants lacking ankyrin-binding. Dileucine mutation, but not loss of ankyrin binding, prevented transcytosis of apically mis-sorted E-cadherin to the lateral membrane. Finally, neurofascin, which binds ankyrin but lacks dileucine residues, exhibited partial apical-lateral polarity that was abolished by mutation of its ankyrin-binding site but was not affected by clathrin knockdown. The polarity motif thus integrates complementary activities of lateral membrane retention through ankyrin-G and apical-lateral transcytosis of mis-localized protein through clathrin. Together, the combination of retention and editing function to ensure a high fidelity steady state localization of E-cadherin at the lateral membrane.

## Teaser

**Background:** E-cadherin targets to the epithelial lateral membrane.

**Results:** Ankyrin-G and clathrin cooperate to control E-cadherin localization.

**Conclusion:** A multifunction motif on E-cadherin interacts with multiple pathways.

**Significance:** This finding provides novel insights into how basolateral targeting motifs function.

## Introduction

Epithelial cells provide the interface between our bodies and the external environment and are critical for ion homeostasis, prevention of microbial invasion, and absorption and excretion of molecules and water. Most epithelial cells are organized in polarized monolayers with apical surfaces directed toward a lumen, lateral membrane domains engaged in cell-cell contact, and basal surfaces associated with extracellular matrix components. Monolayer organization is disrupted during physiological events including wound healing as well as in pathological conditions such as malignant transformation. Given the central role of epithelial tissues in human physiology and disease, it is not surprising that epithelial cell polarity has been the focus of vigorous research over the last three decades (1).

An important clue to the molecular basis for epithelial cell polarity came with discovery of basolateral targeting motifs in cytoplasmic domains of the polymeric immunoglobulin receptors and LDL receptors (2–4). Targeting motifs have subsequently been identified in cytoplasmic domains of many membrane proteins (5). Basolateral targeting signals have been proposed to function through interaction with clathrin adaptors based on similarities with endocytosis signals (3, 5) as well as discovery of an epithelial-specific clathrin AP1 adaptor subunit termed μ1B (6, 7). Moreover, clathrin itself (8) as well as clathrin AP1a and AP1b adaptors (9–11) are required for polarized sorting of certain lateral membrane proteins in MDCK^2^ cells.

Many of the studies on the role of clathrin and its adaptors have utilized proteins such as the LDL receptor and transferrin receptor that experience endocytosis as an essential aspect of their biological function (8, 11). The VSV-G viral coat protein also has frequently been used as a model to study polarity (12). However, it is not clear how the clathrin system contributes to polarity of native proteins that primarily reside in the lateral membrane. For example, nearly complete loss of clathrin results in either partial mislocalization of E-cadherin or no detectable effect on the Na/K-ATPase in MDCK cells (8). Moreover, E-cadherin and Na/K-ATPase both sort to the lateral membranes of LLC-PK cells lacking AP1B, suggesting the existence of an AP1B-independent mechanism (13, 14). AP1A has been recently been shown to be able to compensate for the loss of AP1B (11). However, the lack of effect on Na/K-ATPase and modest effect on E-cadherin localization demonstrated in clathrin heavy chain knockdown cells (8) suggests a role for clathrin-independent mechanisms of lateral membrane localization. Furthermore, in addition, knock-out of either the μ1B subunit or Rab8, which regulates AP1B-dependent basolateral transport, does not affect epithelial morphology in mouse tissues (15, 16). Finally, the Na/K-ATPase segregates into distinct post-Golgi transport intermediates from the AP1B-dependent viral VSV-G protein and does not appear in the recycling compartment (17).

E-cadherin contains an atypical dileucine motif in a juxtamembrane sequence that mediates clathrin-dependent endocytosis (14, 18). The dileucine motif also promotes basolateral targeting of an interleukin-2α receptor-E-cadherin cytoplasmic domain chimera in both MDCK cells and as well as LLC-PK1 cells lacking the AP1B clathrin adaptor (14). However, E-cadherin bearing a dileucine to alanine mutation has been reported by two groups to retain lateral membrane localization (19, 20). The E-cadherin juxtamembrane domain also associates with p120 catenin, which has been proposed to compete for clathrin-mediated endocytosis (21, 22). The juxtamembrane domain in addition is phosphorylated at two tyrosine sites that recruit an E3 ubiquitin ligase termed Hakai that promotes E-cadherin endocytosis (23). However, the roles of p120 and ubiquitination in regulation of E-cadherin apical-lateral polarity are not known.

E-cadherin and Na/K-ATPase both bind directly to ankyrin-G (19, 24–26), which is localized to lateral membranes of epithelial cells, where it is associated with the spectrin-actin network through binding to β2 spectrin (19, 24, 27, 28). The ankyrin-G-spectrin network could contribute to steady state polarity of E-cadherin and Na/K-ATPase through retention of these proteins at the lateral membrane after delivery by either clathrin-dependent or other mechanisms. Ankyrin-G and β2 spectrin may also perform additional roles in basolateral transport of E-cadherin, as knockdown of these proteins results in accumulation of E-cadherin in an intracellular compartment partially co-localizing with the trans-Golgi Network (19, 24, 29). However, elimination of ankyrin-G and β2 spectrin also prevents formation of new lateral membrane in cultured epithelial cells after cytokinesis (19, 24, 29). The absence of lateral membrane in ankyrin-G- and β2 spectrin-depleted cells complicates interpretation of functions of these proteins in the cellular localization of E-cadherin.

We have critically addressed the roles of ankyrin-G and clathrin heavy chain in localization of E-cadherin to the lateral membrane of epithelial cells and observed that E-cadherin retains at least partial polarity after depletion of either ankyrin-G or clathrin heavy chain alone. We find that optimal E-cadherin apical-basal polarity requires both ankyrin-G- and clathrin-based pathways and that these interactions are coordinated by the same highly conserved juxtamembrane motif in the E-cadherin cytoplasmic domain.

## EXPERIMENTAL PROCEDURES

### 

#### 

##### Reagents, Plasmids, and Antibodies

Plasmids encoding E-cadherin (19) and HA-neurofascin-186 (30) were cloned into pEGFP-N1 using standard techniques. An extracellular V5 epitope was inserted into the extracellular domain of E-cadherin in the region between cadherin repeats 3 and 4 using QuikChange II XL mutagenesis (Agilent Technologies). Mutation of the cytoplasmic domain of E-cadherin was also performed using the QuikChange II XL mutagenesis kit according to the manufacturer's instructions. Plasmids encoding pLKO Tet-On were obtained from Addgene (31, 32) and modified as previously described (33).

Mouse anti-E-cadherin (1:1000) and mouse anti-clathrin (1:500 immunostaining; 1:2000 Western blot) were from BD Biosciences. Rabbit anti-ankyrin-G C-terminal domain antibodies (1:1000 immunostaining; 1:5000 Western blot) were previously described (24). Mouse anti-DsRed (1:1000) was from Clontech. Mouse anti-GAPDH (1:5000), rat anti-E-cadherin (DECMA-1, 1:200), and chicken anti-GFP (1:100 after preclearing) were from Abcam. Rabbit anti-mouse IgG was from Pierce (1:5000). Mouse anti-V5 (1:250) as well as all AlexaFluor-conjugated secondary antibodies (1:250) and streptavidin-conjugated AlexaFluor 488 (1:250) were from Invitrogen.

##### Generation of Isoform-specific Ankyrin G Null Mouse

A conditional knock-out mouse was made to delete the Ank3 gene in a tissue-specific manner using the Cre/Lox system. Exons 22 and 23, which are just upstream of the spectrin binding domain, were flanked by LoxP sites. A neomycin resistance cassette, driven by the phosphoglycerate promoter (PGK-neo) and flanked by FRT sites, was inserted between Exon 23 and the 3′ LoxP site. The linearized construct was introduced into 129X1/SvJ ES cells by electroporation, and G418-resistant clones were screened using Southern blot analysis. ES cells bearing the modified Ank3 gene were injected into C57BL/6J blastocysts. High percentage chimeric animals were obtained and bred to C57BL/6 mice to produce heterozygous animals. The PGK-neo cassette was removed by crossing the Ank3flox mice to the mice expressing FLP recombinase (129S4/SvJaeSor-Gt(ROSA)26Sortm1(FLP1)Dym/J, stock number 003946, The Jackson Laboratory). Exons 22 and 23 were excised by crossing the Ank3 flox mouse with the β-actin-Cre mouse (FVB/N-Tg(ACTB-cre)2Mrt/J, stock number 003376, The Jackson Laboratory). Ankyrin-G^−/−^ mice were then generated by crossing ankyrin-G^−/+^ breeders. Generation of target construct, ES cell electroporation, and colony selection, injection of blastocysts, and generation of chimeric mice were provided as a service of the Duke Transgenic Mouse Facility. All experiments were performed in accordance with the guidelines for animal care of the Animal Care and Use Program at Duke University.

##### Immunohistochemistry and Immunocytochemistry

Neonatal mouse pups (p0-p1) were sacrificed by decapitation, and organs were immediately removed and fixed overnight in 4% paraformaldehyde followed by an automated standard overnight paraffin preparation protocol (PBS wash followed by dehydrations through 70, 95, and 100% ethanol with final incubations in xylene and hot paraffin under vacuum). Final paraffin embedding was completed the following day. Paraffin sections were cut at 7 μm using a Leica RM2155 microtome. To ensure identical sample location between mice, kidney sections were taken sagitally at the kidney midline, and trachea sections were taken immediately before the trachea bifurcation into two bronchi. Sections were deparaffinized and rehydrated using a standard protocol of washes: 3 × 3-min xylene washes, 3 × 2-min 100% ethanol washes, and 1 × 2-min 95, 80, and 70% ethanol (each) followed by at least 5 min in PBS. Sections were then processed for antigen retrieval using 10 mm sodium citrate in the microwave for 20 min. Sections were allowed to cool, washed in PBS, and blocked using the Vector MOM kit (Vector Laboratories) according to the manufacturer's protocol. Slides were incubated overnight at 4 °C with primary antibodies diluted in blocking buffer (3% fish oil, 10% horse serum, 0.1% Tween in PBS). After extensive PBS washing, slides were incubated in blocking buffer with the appropriate secondary antibodies for 1 h at room temperature, washed, and mounted with Prolong Gold antifade reagent (Invitrogen).

Cells processed for immunohistochemistry were fixed in 4% paraformaldehyde for 15 min at room temperature and permeabilized with ice-cold methanol for 7 min at −20 °C. Cells were blocked in blocking buffer and incubated overnight at 4 °C with primary antibodies diluted in blocking buffer. After extensive PBS washing, slides were incubated in blocking buffer with the appropriate secondary antibodies for 1 h at room temperature, washed and mounted with Vectashield (Vector Laboratories).

##### Cell Culture and Transfections

MDCKII, HEK293, and HEK293T/17 cells were obtained from the American Type Culture Collection and maintained in a humidified environment at 37 °C with 5% CO_2_. Cells were cultured in DMEM (Invitrogen #11995) with 10% fetal bovine serum, 100 units/ml penicillin, and 100 units/ml streptomycin.

##### Doxycycline-inducible shRNA Cell Lines and Lateral Membrane Biogenesis

Inducible shRNA expression to achieve silencing of ankyrin-G was previously described (33). Briefly, shRNA oligos against the ankyrin-G membrane binding domain (gctagaagtagctaatctcct; targets 190/220-kDa isoforms, but not those lacking ankyrin repeats), clathrin (aatggatctctttgaatacgg), or luciferase control (ggagatcgaatcttaatgtgc) were cloned into pLKO-2A peptide-mCherry. Lentiviruses were generated using psPAX2 and pMD2.G and harvested by centrifugation at 20,000 × *g* for 4 h. MDCK cells were infected with lentivirus in the presence of 8 μg/ml Polybrene overnight. After 48 h, cells were sorted by mCherry fluorescence using fluorescence-activated cell sorting (FACS).

For lateral membrane biogenesis, stable cells lines were preinduced at confluence for 48 h with 5 μg/ml doxycycline, then trypsinized and plated at confluence in 14-mm insert MatTek plates. Cells were fixed at the indicated times and processed for immunocytochemistry. Parallel samples were also prepared for Western blot analysis.

##### Immunoblots

Samples (10-μl volume) were run on a 3.5–17.5% gradient gel in 1× Tris buffer, pH 7.4 (40 mm Tris, 20 mm NaOAc, and 2 mm NaEDTA) with 0.2% SDS (49). Transfer to nitrocellulose was performed overnight at 300 mA at 4 °C in 0.5× Tris buffer with 0.01% SDS. Membranes were blocked with Blot buffer I (150 mm NaCl, 1 mm NaN_3_, 1 mm EDTA, 0.2% Triton X-100, and 10 mm phosphate buffer, pH 7.4) with 2% bovine serum albumin and incubated overnight at 4 °C with primary antibodies diluted in blocking buffer. For experiments involving mouse primary antibodies, membranes were washed with blot buffer I and incubated in rabbit anti-mouse IgG for 1 h at room temperature. Membranes were then incubated with I^125^–labeled protein A/G (1:1000). Membranes were placed on a storage phosphor screen, and signal was detected using a Typhoon imager (GE Healthcare).

##### Apical Mislocalization

MDCK cells grown on MatTek plates were transfected with 50 ng of cDNA encoding V5-E-cadherin-GFP or mutants using Lipofectamine 2000 (Invitrogen) according to the manufacturer's protocol. After 5 h of transfection, cells were fed, and doxycycline (or vehicle control for uninduced samples) was added to the medium at a concentration of 5 μg/ml. 48 h after transfection cells were fixed and prepared for immunocytochemistry as described above. Confocal stacks of MDCKII cells were captured using a Zeiss LSM 780 using a 100 × 1.45 PlanFluor oil objective with 0.25-micron Z spacing and pinhole set to 1 Airy unit. A three-dimensional region corresponding to the apical surface or lateral membrane was drawn using Volocity software (PerkinElmer Life Sciences), and mean pixel intensity was quantified and expressed as mean pixel intensity on the apical membrane *versus* mean pixel intensity on the lateral membrane.

##### Apical E-cadherin Editing

To monitor the fate of apically localized E-cadherin, MDCK cells grown on Transwell filters (Corning) were transfected with 50 ng of cDNA encoding V5-E-cadherin-GFP using Lipofectamine 2000 (Invitrogen) according to the manufacturer's protocol. 48 h after transfection, cells were chilled on ice to minimize protein trafficking and incubated on the apical surface with mouse anti-V5 antibodies in DMEM for 1 h on ice to mark apically localized protein. Cells were quickly washed in cold DMEM and warmed to 37 °C for the indicated times to allow protein trafficking. Cells were fixed in 4% paraformaldehyde for 15 min at room temperature and permeabilized with ice-cold methanol for 7 min at −20 °C. Samples were then incubated with chicken anti-GFP antibodies to mark the total E-cadherin population. Cells were stained with secondary antibodies and mounted as described under “Immunohistochemistry and Immunocytochemistry” above. To aid in visualization, laser settings were adjusted so that the signal intensities for the apically marked protein were similar, as wild-type exhibits much lower levels on the apical surface.

For relocalization of endogenous E-cadherin, MDCK cells grown on Transwell filters were stained on ice with antibodies to the extracellular epitope of E-cadherin (rat E-cadherin) and processed as described above. Total E-cadherin was marked by staining with antibodies to a cytoplasmic epitope (mouse anti-E-cadherin) after fixation and permeabilization.

##### Fluorescence Recovery after Photobleaching

MDCKII cells grown on MatTek plates were transfected with 300 ng of plasmid encoding E-cadherin-GFP or cytoplasmic mutants. 48 h later cells were imaged using a Zeiss Live Duo using a 63 × 1.4 Plan Apochromat oil objective. Three prebleach images were collected in a medial plane, and a 3 × 3-μm region of interest was bleached using high intensity 488-nm illumination. Fluorescence recovery was monitored every 5 s for 300s. Fluorescence intensity within the region of interest was background-subtracted and normalized to a non-bleached region of an adjacent cell to control for photobleaching. Fluorescent recovery after photobleaching (FRAP) curves were fit using a single exponential curve using Graphpad Prism 6. For kymograph analysis, a line was drawn along the lateral membrane, and the Multiple Kymograph plugin was used to generate a kymograph using ImageJ (National Institutes of Health).

##### Membrane Recruitment Assay

The HEK membrane recruitment assay has been previously described (30). Briefly, 1 × 10^5^ HEK293 cells were plated in 14-mm insert, collagen-coated MatTek plates. The following day cells were co-transfected with 100 ng of HA-tagged neurofascin or DsRed-E-cadherin cDNA and 80 ng of GFP-tagged 190-kDa ankyrin-G cDNA using Lipofectamine 2000 according to manufacturer's protocol. 24 h later the cells were fixed and processed for immunofluorescence as described under “Immunohistochemistry and Immunocytochemistry.” The membrane-to-cytoplasm ratio was generated by utilizing line fluorescence intensity analysis of a line drawn across the membrane and cytosol and not including the nucleus of a single medial confocal plane. The membrane peak fluorescence intensity was compared with the mean fluorescence intensity of the entire cytoplasm.

## RESULTS

### 

#### 

##### Selective Knock-out of Large Ankyrin-G Polypeptides in Mice

**FIGURE 1.**
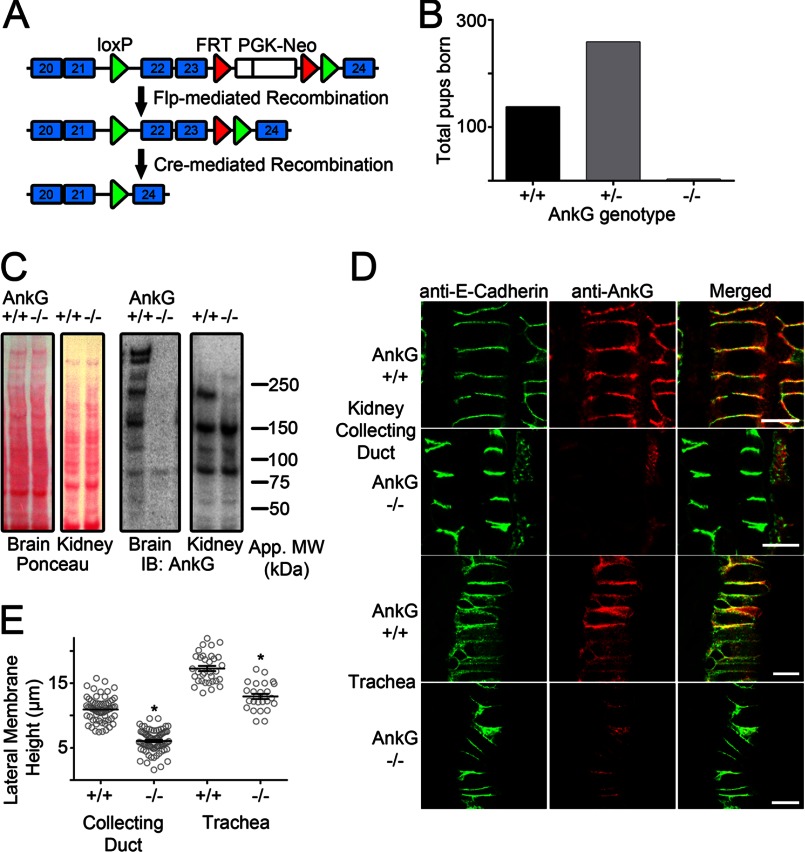
**Selective knock-out of large spliced variants of ankyrin-G in mice results in reduced lateral membrane height but retention of E-cadherin.** *A*, shown is a schematic depiction of the Cre-mediated ankyrin-G null mouse strategy. LoxP sites (*green*), FRT sites (*red*), and a PGK-neo cassette (*white*) were inserted on either side of exons 22 and 23 of mouse ANK3. FLP-mediated recombination was used to remove the PGK-Neo cassette. Cre-mediated excision of exons 22–23 was performed by crossing the floxed mice with the β-actin promoter-driven Cre mouse line. *B*, the total number of ankyrin-G pups born based on genotype (*n* = 138 for +/+; *n* = 259 for +/−; *n* = 3 for −/−). *C*, *left*, shown is Ponceau staining of membranes from wild-type (+/+) or ankyrin-G-null (−/−) littermates from p1 brains or kidneys. *Right*, shown are Western blots (*IB*) of wild type (+/+) or ankyrin-G-null (−/−) littermates from p1 brains or kidneys using an antibody that recognized the ankyrin-G C terminus. *D*, shown are representative images of neonatal mouse kidney collecting duct epithelial cells and trachea epithelial cells stained with anti-E-cadherin to mark the lateral membrane (*green*) and anti-ankyrin-G (*red*). *Bars* represent 10 μm. *E*, shown is quantification of neonatal mouse kidney collecting duct epithelial and trachea epithelial lateral membrane height from wild-type (+/+) or ankyrin-G-null (−/−) littermates. *, *p* < 0.0001 compared with wild-type (Student's *t* test, *n* = 66 for wild-type collecting duct and 84 for ankyrin-G-null collecting duct; *n* = 33 for wild-type trachea and 26 for ankyrin-G-null trachea).

To address the role of ankyrin-G in polarized localization of E-cadherin *in vivo*, we created a floxed mouse that specifically targeted 190-, 270-, and 480-kDa isoforms of ankyrin-G. To achieve a restricted knockdown, we targeted exons 22 and 23 (Fig. 1*A*), which are just upstream of the spectrin binding domain of ankyrin-G and were expected to eliminate spliced forms containing the ankyrin repeats that are required for membrane protein interactions. As an initial test of effectiveness of Cre-mediated ankyrin-G knockdown, we generated β-actin-Cre/floxed ankyrin-G mice, which were expected to exhibit generalized loss of large ankyrin-G polypeptides and suffer prenatal lethality. Most homozygous mutant mice did die before birth on a more pure C57Blk6 background (Fig. 1*B*). Unexpectedly, some ankyrin-G mutant mice survived to birth on a mixed S129/C57Blk6 background, although none survived after postnatal day 2. However, we were able to obtain a few postnatal mice for analysis. β-Actin-Cre/floxed ankyrin-G mice exhibited complete loss of 190-, 270-, and 480-kDa ankyrin-G polypeptides in neonatal brain, indicating the effectiveness of the flox design for these large ankyrin-G polypeptides (Fig. 1*C*, *left*). However, neonatal kidney exhibited loss of 190-kDa ankyrin-G but persistence of polypeptides of 150, 120, and 90 kDa (Fig. 1*C*, *right*). Our strategy of placing flox sites flanking exons 22 and 23 thus unexpectedly spared a tissue-specific subset of ankyrin-G spliced forms that persist in the kidney and likely other tissues. The 150-kDa ankyrin-G polypeptide is large enough to contain ankyrin repeats and may be responsible for survival of these animals to birth. In addition, a 119-kDa ankyrin-G isoform reported in kidney also retains ankyrin repeats but lacks the C-terminal domain recognized by our antibody (34).

Some cell types within the kidney retained membrane-associated ankyrin-G immunoreactivity, suggesting that the shorter, persistent splice isoforms may contribute to lateral membrane formation and maintenance. We focused on the collecting duct epithelial cells because these cells showed an almost complete loss of ankyrin-G immunoreactivity associated with the lateral membrane (Fig. 1*D*). Ankyrin-G-deficient collecting duct epithelial cells demonstrated significantly reduced lateral membrane height from 11 to 5 μm (Fig. 1*E*). Unlike previous work in cells with a complete ankyrin-G knockdown and accompanying loss of lateral membrane (19), we did not detect an obvious internalized population of E-cadherin. This discrepancy between cultured cells lacking a lateral membrane and the animal model may reflect the ability of E-cadherin to properly target to the remaining lateral membrane. In addition, bronchial epithelial cells, which showed a partial loss of ankyrin-G immunoreactivity at their lateral membranes (Fig. 1*D*), also exhibited reduced lateral membrane height from 17 to 12 μm (Fig. 1*E*). These results demonstrate that 190-kDa ankyrin-G is required for normal lateral membrane biogenesis *in vivo* but is not required for localization of E-cadherin to the lateral membranes of either kidney collecting ducts or bronchial epithelial cells. We cannot exclude compensation by other ankyrin-G isoforms that are still expressed in these kidneys or ankyrin-R (Fig. 1 (34)).

##### Comparison of Knockdown of Ankyrin-G and Clathrin in MDCK Cells

**FIGURE 2.**
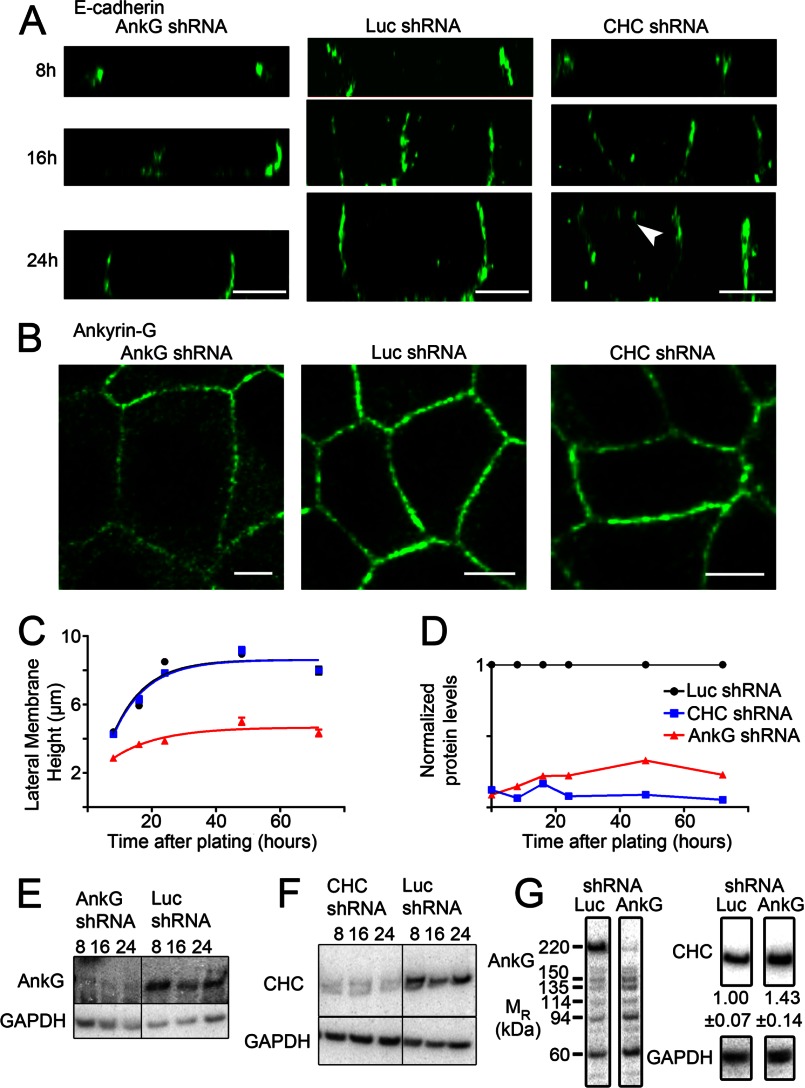
**Lateral membrane biogenesis in MDCK cells requires ankyrin-G but not clathrin.** *A*, shown are representative XZ projections of MDCK cells stably expressing inducible shRNA against ankyrin-G (*left*), luciferase (*middle*), or clathrin (*right*). Cells were plated at high confluence 2 days after induction of shRNA expression and fixed at the indicated times. E-cadherin, marking the height of the lateral membrane, is shown in *green*. The *arrowhead* marks apically mislocalized E-cadherin. *Bars* represent 5 μm. *B*, shown are representative XY images of MDCK cells stably expressing inducible shRNA against ankyrin-G (*left*), luciferase (*middle*), or clathrin (*right*). Cells were plated at high confluence 2 days after induction of shRNA expression and fixed at the indicated times. Ankyrin-G is shown in *green. Bars* represent 5 μm. *C*, shown is the average MDCK lateral membrane height for cells stably expressing inducible shRNA against ankyrin-G (*red triangle*), luciferase (*black circle*), or clathrin (*blue squares*). Average data are shown from 50 cells per time point. *D*, quantification of Western blot for ankyrin-G (*red triangles*) or clathrin (*blue squares*) demonstrate efficient silencing of both ankyrin-G and clathrin. Each time point was normalized to the respective Luc shRNA control. *E*, shown is a Western blot of ankyrin-G in either ankyrin-G shRNA MDCK cells (*left*) or Luc shRNA MDCK cells (*right*) at 8, 16, or 24 h post-plating. GAPDH is used as a loading control. *F*, shown is a Western blot of clathrin in either clathrin (*CHC*) shRNA MDCK cells (*left*) or Luc shRNA MDCK cells (*right*) at 8, 16, or 24 h post-plating. GAPDH was used as a loading control. *G*, shown are Western blots of luciferase shRNA or ankyrin-G shRNA MDCK cells. Labeling with ankyrin-G antibodies demonstrates that only the 220-kDa isoform of ankyrin-G is affected by this shRNA. Migration rate (*M_R_*) is shown in kDa. Antibodies against clathrin heavy chain demonstrate up-regulation of clathrin in ankyrin-G shRNA cells (1.00 ± 0.07 for Luc shRNA and 1.43 ± 0.14 for ankyrin-G shRNA; *n* = 3 for each condition; *p* < 0.05). GAPDH antibodies were used for the loading control.

The persistence of E-cadherin polarity in collecting duct and bronchial epithelial cells partially depleted of ankyrin-G suggested the possibility of a redundant mechanism involving other proteins. Clathrin has been implicated in epithelial polarity in MDCK cells, although the effects of clathrin depletion on E-cadherin localization are modest (8). We, therefore, compared the effects of knockdown of either ankyrin-G or clathrin on both lateral membrane biogenesis and E-cadherin polarity using MDCK cells (Fig. 2). Complete loss of ankyrin-G prevents formation of new lateral membrane after cytokinesis in cultured epithelial cells (24, 29). However, the absence of a lateral membrane complicates interpretation of polarity experiments as proteins may not target properly simply due to the absence of an acceptor membrane. We, therefore, created an inducible shRNA system that allows selection of transfected cells by FACS before induction of shRNA expression. We devised a vector based on pLKO but substituted a viral 2A peptide-mCherry for the IRES-puromycin resistance gene. The 2A peptide mediates co-translational cleavage and allows efficient expression of both mCherry and the Tet repressor. The resulting fluorescent transfected cells can be selected by FACS under non-induced conditions for higher levels of shRNA expression.

MDCKII cells stably expressing inducible shRNA against ankyrin-G, clathrin heavy chain, or luciferase as a control were pre-induced at confluence for 48 h followed by trypsinization and replating at high density to avoid progression through cytokinesis. Under these conditions, ankyrin-G was reduced about 90% (Fig. 2, *D* and *E*). However, the 10% residual ankyrin-G remained associated with the lateral membrane (Fig. 2*B*). Reduction of ankyrin-G in MDCK cells recapitulated results from *in vivo* knockdown with respect to lateral membrane formation and E-cadherin polarity (Fig. 1). Ankyrin-G-depleted cells exhibited a marked reduction in growth of the lateral membrane that remained at about 4 μm after 72 h in culture. In contrast, luciferase shRNA-expressing control cells rapidly assembled lateral membranes and reached an average height of ∼8 μm within 24 h (Fig. 2, *A–C*). However, E-cadherin co-localized with residual ankyrin-G at the remaining lateral membrane in ankyrin-G-depleted cells and did not exhibit intracellular accumulation or apical mis-localization (Fig. 2, *A* and *B*). These results indicate that previously observed internalized E-cadherin in epithelial cells depleted of ankyrin-G and β-2 spectrin (19, 24, 29) was secondary to loss of lateral membrane rather than a specific failure of E-cadherin transport. Interestingly, depletion of ankyrin-G caused an ∼40% increase in clathrin heavy chain levels (Fig. 2*G*). This increase in clathrin may represent a functional coupling between the ankyrin-G and clathrin systems and could compensate for loss of ankyrin-G.

Cells expressing shRNA against clathrin heavy chain, despite >90% reduction of polypeptide levels (Fig. 2, *D* and *F*), were indistinguishable from control cells in the rate or extent of lateral membrane assembly (Fig. 2, *A–C*). However, clathrin-depleted cells did demonstrate modest apical mislocalization of E-cadherin (Fig. 2*A*) consistent with previous reports (8). These results demonstrate that ankyrin-G, but not clathrin, promotes biogenesis of the epithelial lateral membrane and confirm previous observations of partial mis-localization of E-cadherin in the absence of clathrin (8).

##### A Multi-function Motif Determines E-cadherin Polarity

**FIGURE 3.**
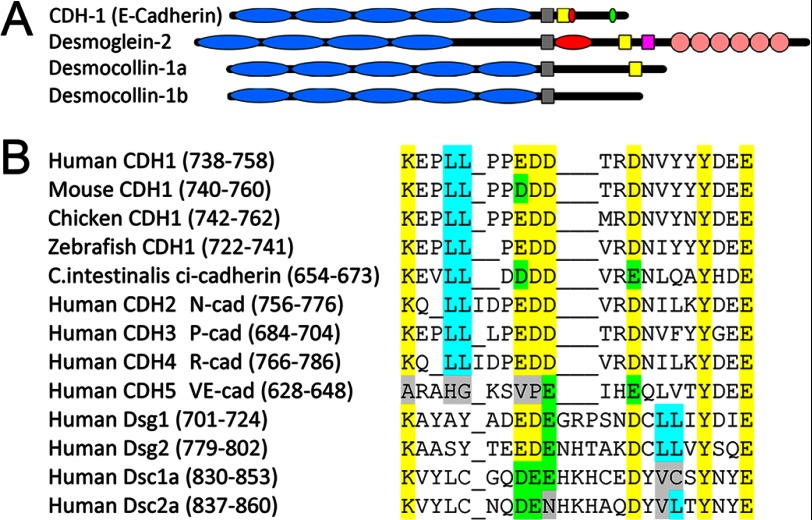
**Identification of a highly conserved, independently evolved motif.** *A*, shown is a schematic depiction of the location of protein interaction sites on cytoplasmic domains of different members of the cadherin superfamily. The transmembrane segment is depicted as a *gray box*. Conserved putative ankyrin-G binding motif is shown as a *yellow box*. The p120 site is shown as a *red ellipse*. The β-catenin binding site is shown as a *green ellipse*. The plakoglobin-binding site is shown as a *magenta box*. Cadherin repeats are shown as *blue ellipses*, and desmoglein repeats are shown as *pink circles. B*, alignment of the proposed ankyrin-G-binding site shows conservation of critical binding residues (*yellow*) both across species and throughout other members of the cadherin super family. The dileucine motif is marked in *cyan*. Conservative substitutions are shown in *green*. Non-conservative substitutions are shown in *gray*.

We next addressed the E-cadherin sequence requirements for polarity. Interestingly, a previously identified atypical dileucine motif resides within the proposed ankyrin-G-binding site (Fig. 3, *A* and *B*). These dileucine residues are required to direct interleukin receptor-E-cadherin chimeras to the lateral membrane (14). However, mutation of the dileucine residues to alanine had no effect on binding of the E-cadherin cytoplasmic domain to ankyrin-G and a small effect on lateral membrane localization of full-length E-cadherin (19, 20).

**FIGURE 4.**
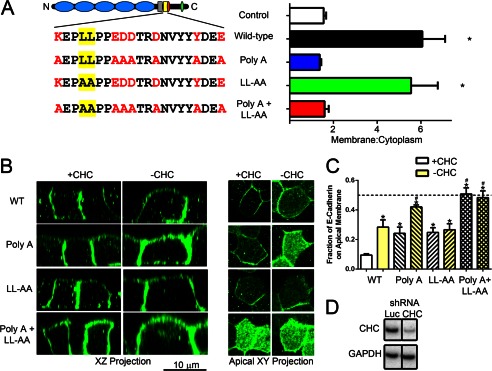
**Clathrin and ankyrin-G cooperate in localization of E-cadherin to MDCK cell lateral membranes.** *A*, *left*, shown is a schematic representation of E-cadherin. Cadherin repeats (*light blue*), transmembrane segment (*gray*), ankyrin-G-binding site (*yellow*), p120 site (*red*), and β catenin site (*green*). Ankyrin-G binding sequences are shown below for wild-type (*top*), Poly(A) (*2nd row*), LL-AA (*3rd row*), and Poly(A) + LL-AA mutant (*bottom*) E-cadherin. Residues critical for ankyrin-G binding are shown in *red*, and the dileucine motif is highlighted in *yellow. Right*, membrane recruitment of ankyrin-G in HEK293 cells is shown. Cells were either transfected with ankyrin-G alone (control, *white*) or plus Wild-type (*black*), Poly(A) (*blue*), LL-AA (*green*), or Poly(A) + LL-AA (*red*) E-cadherin. Line fluorescence intensity analysis was performed on a single plane, and peak pixel intensity on the membrane was compared with average pixel intensity in cytoplasm. *, *p* < 0.05 compared with control, Poly(A), and Poly(A) + LL-AA mutant (one way ANOVA followed by the Tukey post hoc test, *n* = 10 cells for each condition). *B*, MDCK cells stably expressing an inducible clathrin shRNA were transfected with wild-type (*top*), Poly(A) (*2nd row*), LL-AA (*3rd row*), or Poly(A) + LL-AA (*bottom*) E-cadherin in the absence (+*clathrin* (+*CHC*)) or presence (−*clathrin*) of doxycycline induction of clathrin shRNA expression. XZ projections shown on the *left*. The *bar* represents 10 μm. XY projection of apical planes are shown on the *right. C*, shown is quantification of the apical mean pixel intensity in the absence (+*clathrin*) or presence (−*clathrin*) of doxycycline induction of clathrin shRNA expression. Mean pixel intensity of a three-dimensional region of interest containing the apical membrane was quantified and compared with a three-dimensional region of interest containing the lateral membrane. *, *p* < 0.05 compared with WT + clathrin. #, *p* < 0.05 compared with Poly(A) +clathrin (one-way ANOVA followed by the Tukey post hoc test, *n* = 5–8 cells for each condition). *D*, shown is a Western blot of MDCK cells stably expressing either luciferase shRNA (*Luc*, *left*) or clathrin shRNA (*right*) demonstrating silencing of clathrin (*top*) with GAPDH used as a loading control (*bottom*).

To dissect the function of ankyrin binding and dileucine residues in more detail, we created mutants of the E-cadherin cytoplasmic domain that targeted the highly conserved residues within the proposed ankyrin-G-binding site defined previously (19) as well as the dileucine residues that were not required for ankyrin-G binding. The amino acids required for ankyrin binding as well as dileucine residues are conserved back to the urochordate *Ciona intestinalis* (Fig. 3*B*). Interestingly, ankyrin binding residues are conserved within other members of the cadherin superfamily, including desmoglein and desmocollin, whereas dileucine residues are in a different location within the same motif. We generated mutants either (*a*) completely lacking ankyrin binding activity (all seven conserved residues mutated to alanine; referred to as the Poly(A) mutant), (*b*) lacking the dileucine motif (dileucine residues mutated to alanine), or (*c*) lacking both ankyrin binding and the dileucine residues (Fig. 4*A*, *left*). Using a plasma membrane recruitment assay in HEK293 cells (30), we found that the Poly(A) mutant of E-cadherin was unable to recruit ankyrin-G-GFP to the plasma membrane, indicating loss of ankyrin-G binding (Fig. 4*A*, *right*; supplemental Fig. 1). In contrast, the LL/AA mutant of E-cadherin was able to recruit ankyrin-G to a level indistinguishable from wild type, indicating that ankyrin-G binding is not affected by the dileucine mutation (Fig. 4*A*, *right*, supplemental Fig. 1). As expected, the Poly(A) + LL/AA mutant was unable to recruit ankyrin-G (Fig. 4*A*, *right*; supplemental Fig. 1).

To address the roles of the ankyrin binding and dileucine residues of E-cadherin and their relationship to clathrin, we expressed these constructs in polarized MDCK cells stably expressing an inducible clathrin heavy chain-shRNA (see Fig. 2). In the absence of doxycycline induction (+*CHC*), wild-type E-cadherin-GFP localizes 90% to the lateral membrane with <10% mislocalizing to the apical membrane (Fig. 4, *B* and *C*), which is similar to results obtained with endogenous E-cadherin using cell surface biotinylation (8). Mutation of either the residues critical for ankyrin-G binding (Poly(A)) or the dileucine motif (LL/AA) each showed a modest 25–30% apical mis-localization (Fig. 4, *B* and *C*). In contrast, mutation of both ankyrin binding and dileucine residues caused a complete loss of polarized localization of E-cadherin with equal distribution on the apical and lateral membranes (Fig. 4, *B* and *C*). These results demonstrate that E-cadherin polarity requires both ankyrin binding and dileucine residues contained in the same linear sequence.

To determine the role of clathrin in interaction with the dileucine motif, we induced clathrin heavy chain shRNA expression with doxycycline for 48 h, which caused greater than a >90% reduction in clathrin levels compared with luciferase controls (Fig. 4*D*). Induction of clathrin shRNA (−*CHC*) resulted in mis-localization of ∼25% of wild-type E-cadherin to the apical membrane, which is similar to the magnitude of E-cadherin mis-localization observed with the E-cadherin LL/AA mutant in control cells. The LL/AA mutant of E-cadherin, however, was insensitive to clathrin silencing and exhibited no additional mis-localization. In contrast, the Poly(A) mutant of E-cadherin became completely depolarized after clathrin depletion (Fig. 4, *B* and *C*). Apical mis-localization found in the LL/AA mutant, therefore, is likely due to a loss of interaction with the clathrin pathway. These results demonstrate that the cytoplasmic domain of E-cadherin contains overlapping codes for interacting directly with ankyrin-G and likely indirectly with clathrin through the clathrin adaptor(s). Loss of either ankyrin binding residues or dileucine residues causes modest effects, whereas loss of both sites causes randomization of E-cadherin localization.

##### Ankyrin Binding Activity of Neurofascin Is Necessary but Not Sufficient for High Fidelity Apical-Basal Polarity in MDCK Cells

**FIGURE 5.**
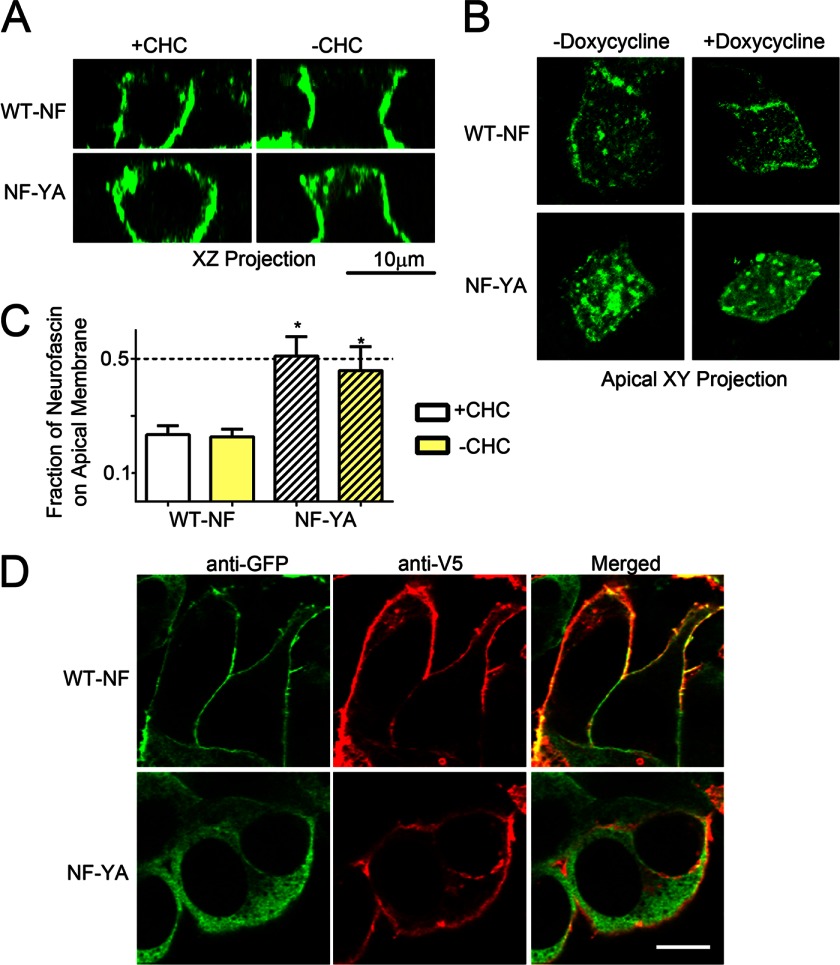
**Ankyrin binding activity of neurofascin is necessary but not sufficient for high fidelity apical-basal polarity in MDCK cells.** *A*, MDCK cells stably expressing an inducible clathrin shRNA were transfected with wild-type neurofascin (*NF*, *top*) or FIGQY-A neurofascin (*NF-YA*, *bottom*) in the absence (+*clathrin* (*CHC*)) or presence (−*clathrin*) of doxycycline induction of clathrin shRNA expression. XZ projections are shown on the left. The *bar* represents 10 μm. *B*, shown is an XY projection of apical planes from cells in *panel A. C*, shown is quantification of apical mean pixel intensity in the absence (+*clathrin*) or presence (−*clathrin*) of doxycycline induction of clathrin shRNA expression. Mean pixel intensity of a three-dimensional region of interest containing the apical membrane was quantified and compared with a three-dimensional region of interest containing the lateral membrane. *, *p* < 0.05 compared with WT + clathrin (one-way ANOVA followed by Tukey post hoc test, *n* = 5 cells for each condition). *D*, shown are representative images of membrane recruitment of ankyrin-G-GFP (*green*) by wild-type V5–186-kDa neurofascin (*WT-NF*) but not V5-FIGQY-A neurofascin (*NF-YA*). The V5 signal is shown in *red*. The merged image is shown on the *right*. The *bar* represents 10 μm.

These results suggest that optimal polarity for E-cadherin requires both ankyrin binding and capability of engaging the clathrin pathway. The E-cadherin juxtamembrane domain also interacts with p120 catenin (21, 35) and possibly other proteins. To further examine the role of ankyrin binding independent of p120 in polarized localization of epithelial proteins, we expressed the 186-kDa form of neurofascin in polarized MDCK cells. Neurofascin tightly binds to ankyrin-G through an independently evolved binding site, and loss of ankyrin-G binding activity through mutation of a critical tyrosine residue causes mis-localization of neurofascin from the axon initial segment (30, 36, 37). Neurofascin, however, does not contain an endocytosis motif and likely does not participate in clathrin-mediated endocytosis. When expressed in MDCK cells, wild-type neurofascin exhibits lateral membrane localization, although the fidelity of this localization is not as great as E-cadherin with ∼25% mislocalization to the apical membrane (Fig. 5). Mutation of the tyrosine within the previously identified ankyrin-G-binding site to alanine (*NF YA*) caused a complete loss of polarized neurofascin localization (Fig. 5). However, silencing of clathrin had no effect on wild-type neurofascin. These results demonstrate that ankyrin-G binding is sufficient for partial lateral membrane localization of neurofascin in polarized MDCK cells but that additional factors are required for complete exclusion from the apical membrane.

##### The Polarity Motif Restricts E-cadherin Mobility within the Lateral Membrane

**FIGURE 6.**
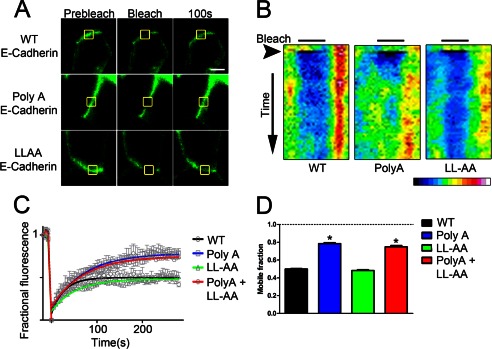
**Poly(A) E-cadherin demonstrates increased mobility within the MDCK lateral membrane.** *A*, representative images of FRAP experiments in MDCK cells transiently expressing Wild-type (*top*), Poly(A) (*middle*), and LLAA (*bottom*) E-cadherin-GFP are shown before (*Prebleach*), immediately after (*Bleach*), or 100 s after high laser power illumination of the bleach region of interest (*yellow square*). The *bar* represents 5 μm. *B*, kymographs are from cells in *panel A*. A line was drawn along the lateral membrane, and fluorescence intensity was quantified along that line from time 0 (*top*) to 300 s (*bottom*). The bleach region of interest is denoted by a *black bar*. Time of bleach is marked by an *arrowhead*. The fluorescence intensity scale is shown below. *C*, shown are FRAP recovery curves for wild type (*black*), Poly(A) (*blue*), LL-AA (*green*), and Poly(A) + LL-AA mutant (*red*) E-cadherin-GFP. Curves represent single exponential best fits of the average data from seven cells per condition. *D*, shown is mobile fraction of FRAP recovery for recovery curves from *panel C*. *, *p* < 0.05 compared with WT and LL-AA E-cadherin (one-way ANOVA followed by Tukey post hoc test, *n* = 7 cells per condition).

To evaluate a possible role of ankyrin binding in retention of E-cadherin to the lateral membrane, we performed FRAP analysis of wild-type and mutant E-cadherin-GFP on the lateral membrane of polarized MDCK cells. After bleaching a region in a medial plane of a MDCK lateral membrane, wild-type E-cadherin achieved a plateau of ∼50% recovery within 5 min (Fig. 6), consistent with a previous report demonstrating a much longer time (>20 min) needed to achieve complete recovery (38). Poly(A)-E-cadherin exhibited a much higher mobile fraction (∼80%), indicating that loss of ankyrin binding removed a restraint limiting lateral mobility of E-cadherin (Fig. 6). In contrast, mutation of the dileucine motif had no effect on FRAP recovery of E-cadherin (Fig. 6). Finally, mutation of both the ankyrin-G binding residues and the dileucine motif showed no additional effect on mobile fraction compared with the Poly(A) mutation alone (Fig. 6). These results show that the Poly(A) mutations, which confer loss of ankyrin-G binding, also cause an increased mobile fraction of E-cadherin within the lateral membrane. This suggests that association with ankyrin-G restricts E-cadherin mobility within the plane of the lateral membrane and could also function as a retention mechanism.

##### Dileucine Mutation Prevents Retrieval of Apically Mis-sorted E-cadherin

**FIGURE 7.**
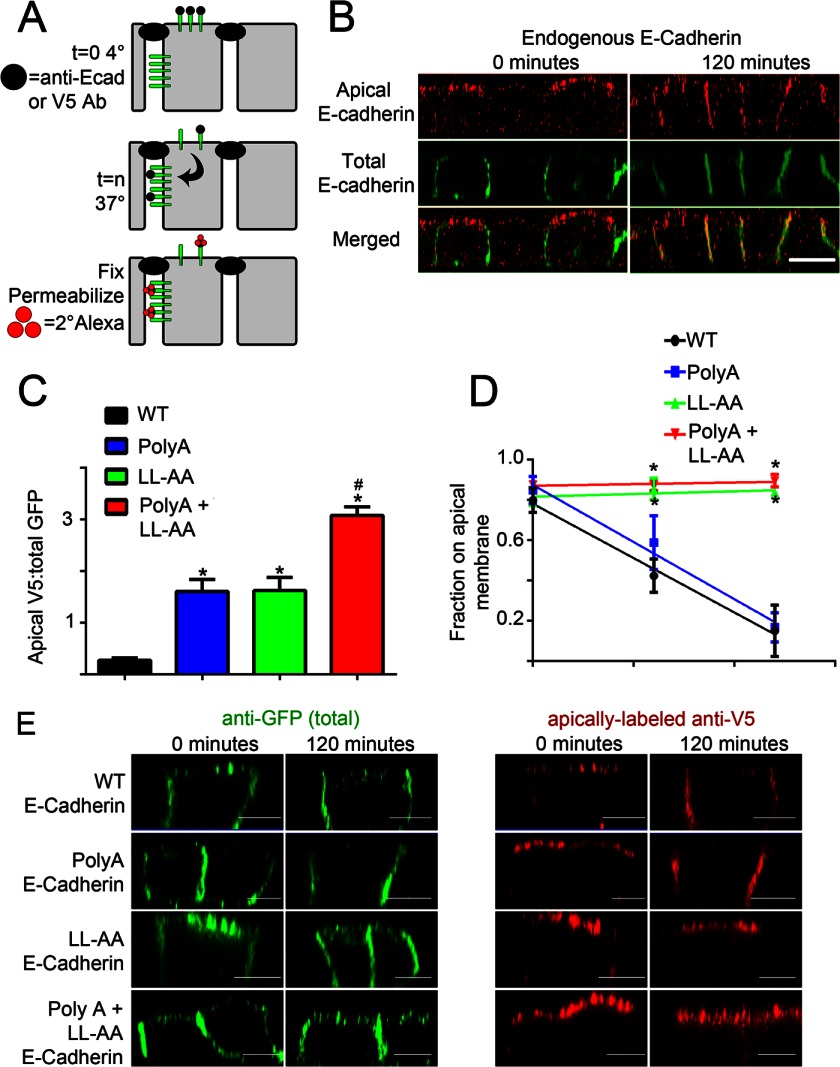
**Dileucine/alanine mutation abolishes editing of apically mislocalized E-cadherin in MDCK cells.** *A*, a model of apical E-cadherin internalization assay is shown. Confluent, filter-grown MDCK cells were transfected with E-cadherin containing an extracellular V5 epitope and a cytoplasmic GFP epitope. Cells were then labeled on the apical surface with primary antibody (*black circle*) on ice for 1 h followed by extensive washing. Cells were warmed to 37 °C for the indicated times to allow trafficking of E-cadherin from the apical membrane. Cells were then fixed, permeabilized, and labeled with secondary antibodies tagged with Alexa568 (*red circles*). *B*, shown are representative XZ projections of MDCK cells stained with antibodies to endogenous E-cadherin. Total E-cadherin is marked with mouse anti-E-cadherin antibodies (*green*). Apically labeled antibody to the extracellular epitope of E-cadherin (rat anti-E-cadherin) is shown in *red. Bars* represent 10 μm. *C*, shown is quantification of the amount of apical V5 labeling of V5-E-cadherin and mutants normalized to total GFP expression. *, *p* < 0.05 compared with wild-type. #, *p* < 0.05 compared with both Poly(A) and LL-AA mutants (one way ANOVA followed by Tukey post hoc test; *n* = 5 cells per condition). *D*, shown is the quantification of the fraction of apically labeled E-cadherin remaining on the apical surface. Mean pixel intensity of a three-dimensional region of interest containing the apical membrane was quantified and compared with a three-dimensional region of interest containing the lateral membrane. *, *p* < 0.05 compared with both wild-type and Poly(A) E-cadherin (two-way ANOVA followed by Tukey post hoc test, *n* = 5 cells for point). *E*, shown are representative XZ projections of MDCK cells transfected with wild-type (*top*), Poly(A) (*2nd row*), LL-AA (*3rd row*), or Poly(A) + LL-AA mutant (*bottom*) E-cadherin warmed to 37 °C for 0 min (*left*) or 120 min (*right*). Total E-cadherin-GFP is marked with anti-GFP antibodies and is shown in *green* (*left*). Apically labeled anti-V5 to the extracellular epitope of E-cadherin is shown in *red* (*right*). *Bars* represent 5 μm.

We next addressed how the dileucine residues contribute to E-cadherin localization. We evaluated the hypothesis that clathrin, acting through its well established role in mediating endocytosis of plasma membrane proteins, removes apically mis-sorted E-cadherin. Native E-cadherin exhibits less than 5% apical label at steady state, which is difficult to detect using standard labeling procedures (Fig. 2*A*). However, apical staining can be amplified using vectorial labeling at 4 ºC from the apical compartment with antibody against the E-cadherin extracellular domain (Fig. 7*A*). Interestingly, native E-cadherin labeled on the apical surface relocates to the lateral membrane over a 2-h period after warming to 37 ºC (Fig. 7*B*). To determine the role of specific cytoplasmic sequences in this relocalization, we introduced an extracellular V5 epitope into wild-type and mutant versions of E-cadherin-GFP, which allowed detection of apically localized E-cadherin by incubation with V5 mouse antibody at 4 ºC (Fig. 7*A*). The extent of apical *versus* lateral E-cadherin was measured in arbitrary units by determining the ratio of lateral label with antibody against GFP to apical label with antibody against mouse IgG (Fig. 7*C*). At the starting time point, before warming, wild-type E-cadherin exhibited low but detectable apical label, whereas both the Poly(A) and LL/AA mutants exhibited 5-fold more apical relative to lateral protein than wild-type (Fig. 7*C*). The Poly(A) + LL/AA mutant exhibited a further 2-fold increase in the apical/lateral ratio or about 10-fold above wild type (Fig. 7*C*). Similar patterns of negligible apical localization for wild type, increased apical localization with Poly(A) and dileucine mutants, and equivalent apical and lateral localization for the double Poly(A) +LL/AA mutants are evident in the steady state experiment in Fig. 4. Importantly, apical labeling with V5 antibody demonstrates that the mislocalized E-cadherin shown in Fig. 4 is indeed on the apical surface and accessible to the extracellular V5 antibody.

We next determined the fate of apically labeled wild type and mutant E-cadherin by warming the cells to 37 ºC for various times followed by fixation, permeabilization, and labeling with a fluorescent anti-mouse IgG (Fig. 7*A*). Apical wild-type E-cadherin completely re-localized to the lateral membrane over the course of 2 h (Fig. 7, *D* and *E*). Similarly, apical Poly(A) E-cadherin mutant also rapidly and nearly completely relocalized to the lateral membrane. However, both the LL/AA and Poly(A) + LL/AA E-cadherin mutants remained at the apical membrane with minimal transcytosis to the lateral membrane (Fig. 7, *D* and *E*). Alanine mutation of the dileucine residues thus prevents retrieval of apically mislocalized E-cadherin and delivery to the lateral membrane.

## DISCUSSION

We report here that a highly conserved 21-amino acid stretch in its cytoplasmic domain determines apical-lateral polarity of E-cadherin and that distinct residues within this motif confer both restricted mobility at the lateral membrane as well as transcytosis of apically mis-sorted proteins to the lateral membrane. Mutations resulting in separate loss of either restricted mobility or apical-lateral transcytosis result in partial mis-sorting. However, loss of both activities results in completely randomized localization of E-cadherin between apical and lateral domains in MDCK cells. The following evidence supports the interpretation that residues required for restricted mobility mediate retention at the lateral membrane through interaction with ankyrin-G, whereas dileucine residues conferring apical-lateral transcytosis act through a clathrin-dependent process that functions as an editing pathway. Ankyrin-G interaction with E-cadherin is abolished by the same mutations that result in increased E-cadherin mobility but not by dileucine mutation, eliminating apical-lateral transcytosis (Figs. 4 and 7). Clathrin knockdown and dileucine mutation both cause the same partial loss of polarity of E-cadherin (Fig. 4). Moreover, clathrin knockdown causes no further change in polarity of E-cadherin with dileucine mutation but completely randomizes E-cadherin mutants with loss of ankyrin binding (Fig. 4). Finally, neurofascin, which has an independently evolved ankyrin binding site but lacks a dileucine motif, exhibited partial apical-lateral polarity that was abolished by mutation of ankyrin-binding site but was not affected by clathrin knockdown.

**FIGURE 8.**
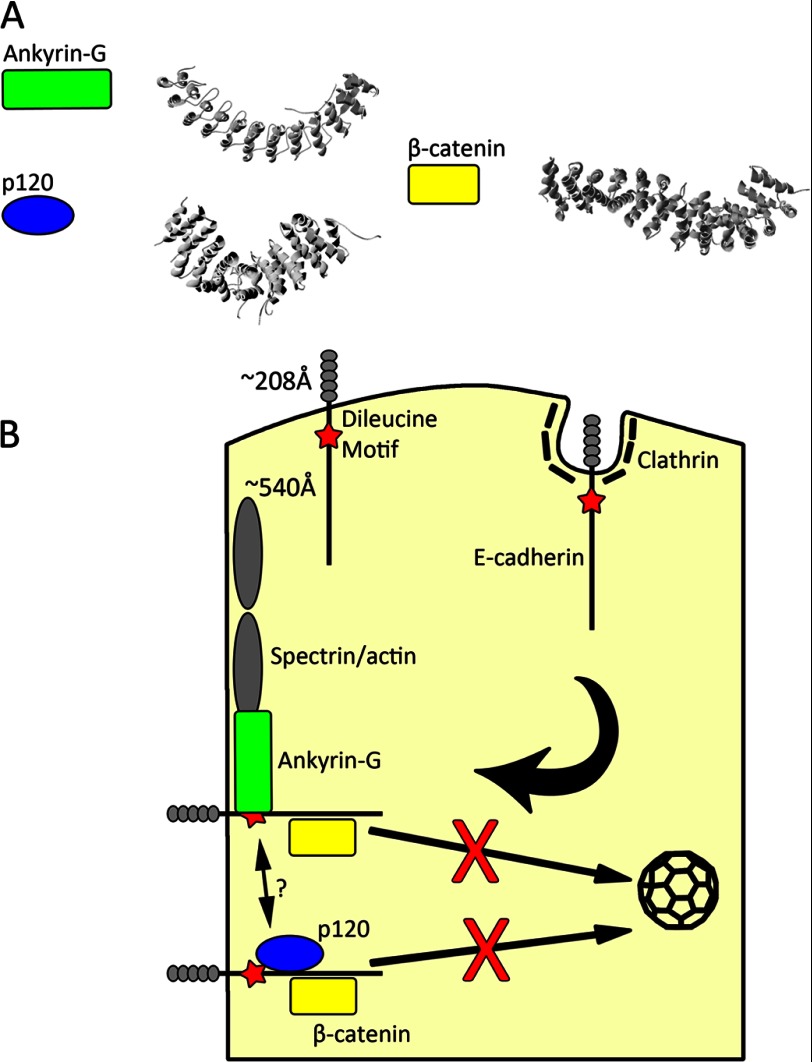
**Schematic model of complementary roles of the E-cadherin polarity motif in lateral membrane retention and apical membrane retrieval.** *A*, ribbon diagrams based on crystal structures from ankyrin-G, p120, and β-catenin (21, 46, 47) show the prevalence of the solenoid structure in binding of the unstructured cytoplasmic domain of E-cadherin. *B*, shown is a model for E-cadherin localization. The dileucine motif (*red star*) engages the clathrin system for internalization of apically mislocalized protein. In contrast, protein localized to the lateral membrane can interact with ankyrin-G (*green rectangle*) and thus the underlying spectrin/actin cytoskeleton, p120 (*blue ellipse*), and or β-catenin (*yellow rectangle*). Interaction with p120, and perhaps ankyrin-G, will sterically hinder access to the dileucine motif and minimize clathrin-mediated endocytosis of properly localized protein. More work remains to be done to determine if ankyrin-G binding precludes binding of p120. Length of domains shown are based on the crystal structure for E-cadherin ectodomain (48) and using 152 amino acids at 3.6 Å per amino acid for cytoplasmic domain.

These findings suggest a model where the E-cadherin polarity motif integrates the distinct but complementary activities of lateral membrane retention through ankyrin-G and apical-lateral transcytosis of mis-localized protein through clathrin. Together the combination of membrane retention and editing functions ensure a high fidelity steady state localization of E-cadherin at the lateral membrane. Steric considerations suggest that the polarity motif can interact with either ankyrin-G or a clathrin-based adaptor but not both at the same time. Thus E-cadherin associated with ankyrin-G on the lateral membrane would not be engaged by the clathrin system, whereas E-cadherin mis-localized to the ankyrin-G-free apical domain would be removed by endocytosis (Fig. 8*B*).

Such a model begs the question of how ankyrin-G itself localizes to epithelial lateral membranes. Several lines of evidence support an intimate relationship between ankyrin-G and the lateral membrane independent of E-cadherin. We demonstrate here that knock-out of 190-kDa ankyrin-G in the mouse kidney collecting duct (Fig. 1) and in MDCK cells (Fig. 2) results in marked reduction in lateral membrane height and rate of lateral membrane biogenesis. Moreover, ankyrin-G persists on the plasma membrane of MDCK cells in low calcium in the absence of E-cadherin and β-2 spectrin (33).

Surprisingly, mutation of the ankyrin-G-binding site in E-cadherin (Fig. 4) caused apical mis-localization that was not seen in either the ankyrin-G mutant mouse (Fig. 1) or in the shRNA experiments in cultured cells (Fig. 2; Ref. 19). Interestingly, shRNA silencing of ankyrin-G causes up-regulation of clathrin heavy chain (Fig. 2*G*). Thus, under conditions of chronic loss of ankyrin-G, clathrin-dependent editing is up-regulated and can promote removal of apically mislocalized proteins. In addition, divergent results involving apical mislocalization of E-cadherin likely reflect the very different types of experiments used to address the role of ankyrin-G. Both the null mouse (∼21 days) and hypomorphic knockdown of ankyrin-G (5 days) are chronic conditions that result in depletion but not complete removal of ankyrin-G. In contrast, the Poly(A) mutation of E-cadherin completely lost interaction with ankyrin-G (Fig. 4*A*) and was transiently expressed in the context of normal levels of wild-type E-cadherin, which may saturate the carrying capacity of the clathrin editing pathway. It also is possible that the residual ankyrin-G, which remains localized at the lateral membrane (Figs. 1 and 3) is sufficient to retain E-cadherin.

Up-regulation of clathrin heavy chain in response to ankyrin-G silencing (Fig. 2*G*) indicates a functional coupling between ankyrin-G and clathrin systems that goes beyond sharing the same linear motif. This result demonstrates that ankyrin-G and clathrin are functionally coupled at the protein level. This result strengthens the model where ankyrin-G and clathrin cooperate to control localization of lateral membrane proteins. Moreover, this cross-talk and compensation may indicate why loss of just one binding site in the polarity motif causes relatively modest effects, whereas loss of both binding activities causes a dramatic loss of polarity.

The E-cadherin polarity motif is preserved in the chordate lineage and is unaltered except for two conservative substitutions as far back as the urochordate *C. intestinalis* (Fig. 3). *C. intestinalis* has only one ankyrin gene (39), suggesting that all three current ankyrins evolved from an ancestral ankyrin with cadherin binding activity. Ankyrin-B and ankyrin-R thus may also have cadherin-binding sites and collaborate with clathrin in establishing localization of members of the cadherin family. It is of interest in this regard that “classical” N-, P-, and R-cadherins have closely related polarity motifs, whereas VE and K-cadherins have divergent sequences (Fig. 3). Desmosomal cadherins (desmoglein 1–4 and desmocollin 1–3) contain ankyrin binding residues, although with dileucines in a different location in the 21-amino acid motif (Fig. 3). The E-cadherin polarity motif is substantially different in hemichordates, echinoderms, mollusks, and *Drosophila* and *Caenorhabditis elegans*. A cadherin-ankyrin-clathrin-based polarity mechanism, thus, is likely a chordate invention and could have many physiological roles in modern vertebrates.

The identity of clathrin adaptor(s) that interacts with the E-cadherin polarity motif and the intracellular route of apically mis-sorted E-cadherin to the lateral membrane both remain to be determined. Clathrin is a major hub in a complex and dynamic interaction network that includes many possible adaptors (40, 41). Possible endocytic routes also are potentially complicated and likely include an endosome recycling compartment intermediate. Thus it is probable that more than one clathrin adaptor and membrane compartment is involved in apical-lateral transcytosis of mis-sorted E-cadherin (11).

The finding that multiple functions are encoded in a single short stretch of amino acids challenges parsimonious assumptions of a single adaptor for basolateral targeting motifs. In addition to the clathrin adaptor(s) and ankyrin-G, the juxtamembrane domain also interacts with p120 catenin (21) and Hakai (after tyrosine phosphorylation) (23). p120 catenin has been proposed to modulate E-cadherin endocytosis from the lateral membrane through competition with clathrin adaptor(s) interacting with the dileucine residues (20, 21, 42). p120 catenin and ankyrin-G also are likely to compete for binding to E-cadherin based on p120-interactions that extend well beyond the core binding site (21). Interestingly, although the p120- and ankyrin-G-binding sites are neighbors in the classical cadherins, they have shifted positions in other members of the cadherin superfamily (Fig. 3*A*), indicating that they are not obligate partners. In addition, neurofascin, which does not contain a p120 binding site, still demonstrate lateral membrane localization (Fig. 5). A function for ankyrin-G binding in desmosomal cadherins remains to be evaluated. The major endothelial cadherin, VE-cadherin, has been recently shown to possess a dual-function motif overlapping with the polarity motif defined here that controls its dynamic behavior (22). A cluster of acidic residues within the VE-cadherin cytoplasmic domain functions as an endocytic signal, which is masked by binding of p120. Interestingly, VE-cadherin has lost both the dileucine residues and the residues critical for ankyrin-G binding found in the classical cadherins (Fig. 3*B*).

The E-cadherin cytoplasmic domain is intrinsically unstructured in solution and thus presents an extensive surface that is actually longer than the extracellular domain (Ref. 43; see Fig. 8 where these domains are drawn to scale). Intrinsically unstructured proteins in general have received much recent attention due to their versatility in rapid evolution of protein binding sites and ability to “moonlight” with multiple partners (44). Ankyrin, p120, and β-catenin are each composed of short repeats that -fold into solenoid-like structures with extended surfaces well suited for association with unstructured peptides that potentially could include membrane proteins in addition to E-cadherin (Fig. 8*A*). It is pertinent in this regard that neurofascin and other ankyrin partners are configured as either unstructured peptides or loops (45). Together these considerations suggest more generally that the apparently simple basolateral targeting motifs actually are sophisticated codes operating in a complex interactome that determine interactions with multiple adaptors and are important sites of regulation.

190-kDa ankyrin-G is required for normal lateral membrane height both *in vivo* and in cultured epithelial cells, suggesting a more global role than just interaction with E-cadherin (Figs. 1 and 2). In cultured cells, complete knockdown of 190-kDa ankyrin-G or β-2 spectrin in pre-polarized cells prevents formation of new lateral membrane during cytokinesis and is accompanied by multinucleated cells and subsequent cell death (24, 29). In addition, complete knockdown results in intracellular accumulation of E-cadherin (19). We, therefore, developed an inducible-knockdown strategy that allowed cells to retain sufficient ankyrin-G to complete cell division and assemble a residual lateral membrane, although subsequent membrane growth was markedly reduced (Fig. 2). Ankyrin-G could contribute to maintenance of the lateral membrane by restricting the lateral mobility and impeding endocytosis of membrane proteins in addition to E-cadherin. It will be important in future work to determine the full scope ankyrin-G interactions with lateral membrane proteins and their basolateral targeting motifs.

## Footnotes

2 The abbreviations used are:

MDCK cells: Madin-Darby canine kidney cells
FRAP: fluorescent recovery after photobleaching
ANOVA: analysis of variance.
